# Improved Molecular Diagnosis of McCune–Albright Syndrome and Bone Fibrous Dysplasia by Digital PCR

**DOI:** 10.3389/fgene.2019.00862

**Published:** 2019-09-18

**Authors:** Francesca Marta Elli, Luisa de Sanctis, Massimiliano Bergallo, Maria Antonia Maffini, Arianna Pirelli, Ilaria Galliano, Paolo Bordogna, Maura Arosio, Giovanna Mantovani

**Affiliations:** ^1^Department of Clinical Sciences and Community Health, University of Milan, Milan, Italy; ^2^Department of Public Health and Pediatric Sciences, University of Torino, Regina Margherita Children’s Hospital-AOU Cittàdella Salute e dellaScienza, Torino, Italy; ^3^Endocrinology Unit, Fondazione IRCCS Ca’ GrandaOspedale Maggiore Policlinico, Milan, Italy

**Keywords:** McCune–Albright’s syndrome, bone fibrous dysplasia, precocious puberty, GNAS, mosaicism, digital PCR

## Abstract

McCune–Albright syndrome (MAS) is a rare congenital disorder characterized by the association of endocrine and nonendocrine anomalies caused by somatic activating variants of *GNAS*. The mosaic state of variants makes the clinical presentation extremely heterogeneous depending on involved tissues. Biological samples bearing a low level of mosaicism frequently lead to false-negative results with an underestimation of causative molecular alterations, and the analysis of biopsies is often needed to obtain a molecular diagnosis. To date, no reliable analytical method for the noninvasive testing of blood is available. This study was aimed at validating a novel and highly sensitive technique, the digital PCR (dPCR), to increase the detection rate of *GNAS* alterations in patients with a clinical suspicion of MAS and, in particular, in blood. We screened different tissues (blood, bone, cutis, ovary, and ovarian cyst) collected from 54 MAS patients by different technical approaches. Considering blood, Sanger was unable to detect mutations, the allele-specific PCR and the co-amplification at lower denaturation temperature had a 9.1% and 18.1% detection rate, respectively, whereas the dPCR reached a 37.8% detection rate. In conclusion, the dPCR resulted in a cost-effective, reliable, and rapid method allowing the selective amplification of low-frequency variants and able to improve *GNAS* mutant allele detection, especially in the blood.

## Introduction

The McCune–Albright syndrome (MAS, MIM #174800) is a rare congenital disorder that comprises the clinical triad of endocrinopathies, cafè-au-lait pigmented skin lesions (SP), and fibrous dysplasia of bone (FD). The endocrine dysregulation may include various autonomous hormonal hyperfunctions, such as precocious puberty, hyperthyroidism, growth hormone excess, adrenal hyperplasia, hypophosphatemic osteomalacia, and GH- and/or PRL- secreting pituitary tumors (McCune, 1936; Albright et al., 1937; Robinson et al., 2016; Boyce et al., 2017). In particular, the more frequent and studied alterations of the endocrine glands are the gonadal hyperfunction (characterized by episodes of hyperestrogenism with a consequent reduction in gonadotropin secretion), thyroid abnormalities (nodular goiter with nodules >1cm), and the GH-IGF1 axis hyperactivity (Matarazzo et al., 2006; Tessaris et al., 2012; Tessaris et al., 2006).

The disease demonstrated sporadic occurrence, phenotypic heterogeneity, variable involvement of different endocrine glands, and a pattern of skin and bone lesions following the lines of the embryologic development, thus it was proposed that MAS derived from a postzygotic genetic defect, leading to a mosaic distribution of mutated cells. Such hypothesis was confirmed by the identification of somatic activating variants affecting the alpha subunit of the stimulatory G protein (Gsα, encoded by GNAS, MIM*139320) (Landis et al., 1989; Weinstein et al., 1991; Schwindinger et al., 1992; Candeliere et al., 1997). Most gain-of-function *GNAS* genetic variants occurred at the Arg202 residue (historically and still frequently reported as Arg201) and determined the constitutional activation of the stimulatory G protein and, consequently, of adenylyl cyclase (Landis et al., 1989). The tissue-by-tissue study by droplet digital PCR (ddPCR) of autopsy samples from a patient confirmed that MAS can affect a wider range of tissues, leading to extraskeletal manifestations, such as gastrointestinal reflux and/or polyps, pancreatitis, hepatobiliary disease, cardiac disease (sudden death, tachycardia, high output heart failure, and aortic root dilatation), platelet dysfunction, and cancer (bone, breast, testes, and thyroid) (Salpea and Stratakis, 2014; Vasilev et al., 2014).

The mosaic state of MAS/FD-associated variants determines an extremely heterogeneous presentation of clinical manifestations in patients, depending upon the extent to which affected tissues are involved, and makes establishing the correct molecular diagnosis a real challenge. As a matter of fact, the investigation of biological samples, such as peripheral blood that typically bears a low level of mosaicism, leads to high rates of false-negative results and the underestimation of causative molecular alterations in MAS/FD patients (Hannon et al., 2003; Karadag et al., 2004; Lumbroso et al., 2004; Lietman et al., 2005; Kalfa et al., 2006; Kuznetsov et al., 2008; Liang et al., 2011; Narumi et al., 2013).

A real help to overcome the underestimation of mosaic variants will possibly arrive by the development of single-cell sequencing technologies that, actually, are under development as a liquid biopsy tool to investigate cancer patients noninvasively. In particular, limits, such as elevated costs and obtaining enough genetic material for library preparation, make these methods still far from the adoption by the health care system, but, in the next future, they could represent a promising tool for somatic variants analysis (Wei et al., 2017; Zhu et al., 2018).

Different technical approaches, each with its pros and cons, had been developed in the past years with the aim to detect MAS/FD-associated variants. The first attempt dates back to 1997 when Candeliere and colleagues, (1997) combined consecutive and repeated cycles of PCR amplification with site-directed mutagenesis to generate a PCR product from the normal allele susceptible to restriction nuclease digestion. Other researchers tried to overcome the problem by using a peptide nucleic acid (PNA) primer to inhibit the amplification of the normal allele, combining PNA probes with the fluorescence resonance energy transfer (FRET) technique, pyrosequencing, next-generation sequencing (NGS), or ddPCR (Hannon et al., 2003; Karadag et al., 2004; Liang et al., 2011; Narumi et al., 2013; Vasilev et al., 2014). Selective enrichment methods reached good levels in low-abundance variants detection, but they presented several limits as being expensive, time-consuming, and leading to an elevated risk of generating PCR artifacts and cross-contamination (Qin et al., 2016). In particular, the NGS approach with deep sequence coverage enhances sensitivity and allows for accurate quantification of the level of mosaicism, but the use of a customized DNA probe library followed by deep NGS analysis with a mean coverage depth per base of approximately 800× translates into a cost-per-patient analysis too high for the health care system.

The improvement in the sensitivity of genetic tests for defects in mosaic state associated with MAS/FD is an important step to improve the diagnosis of patients, thus the aim of the present study was to set up and validate a novel technique, digital PCR (dPCR), to increase the detection rate of activating *GNAS* alterations in different tissue samples. The dPCR is a method used for absolute quantification of nucleic acids based on the amplification of single molecules of template with target-specific fluorescent-labeled assays. The subsequent analysis yields the relative or absolute quantification results from the raw imaging data. It was conceived in 1992 and applied to the quantification of KRAS mutations in DNA from colorectal cancer patients. This approach has many potential applications, including the detection and quantification of low-level pathogens, rare genetic sequences, copy number variations (CNVs), gene expression in single cells, and quantification of circulating miRNA expression. It works by partitioning a sample of DNA or cDNA into many individual parallel PCR reactions and allows the detection of sequence variants present at a very low frequency in a pool of wild-type background (Sykes et al., 1992; Vogelstein and Kinzler, 1999). According to the manufacturer’s specification, optimized dPCR assays should detect and quantify rare mutant prevalence to ≤0.1%.

In this study, we evaluated the usefulness of a novel technique, the dPCR, to obtain a molecular confirmation in patients with a clinical suspicion of MAS/FD by the screening of different tissues collected from a case series of 54 MAS/FD patients using and comparing different technical approaches. In particular, thanks to the expected ability of dPCR to detect low-abundance somatic single-nucleotide alterations, our main aim was to find *GNAS* mutant alleles in blood samples from MAS/FD patients.

## Materials and Methods

### Patients and Biological Samples

The present study included 54 patients with a clinical diagnosis of MAS/FD. For 41 patients, a single tissue only was available for the analysis [24 blood samples (BL) and 17 biopsies: 10 bone (BO), 4 cutis (CB), 3 ovarian tissue (OT), and/or ovarian cyst (OC)], whereas for 13 patients, we investigated and compared the DNA extracted from different tissues (2 BL+BO, 6 BL+CB/fibroblasts from cutaneous biopsy, and 4 BL+OT/OC). Overall, 79 different samples were tested for the presence of MAS/FD-related activating variants within *GNAS* exon 8. The MAS clinical suspicion was based on the presence of at least persistent and/or recurrent ovarian cysts associated with peripheral precocious puberty or another typical sign of MAS between cafè-au-lait skin spots or fibrous bone dysplasia. Clinical details, including some patient-specific additional features, and the molecular diagnosis are resumed in the **Supplementary Table 1**. Wild-type controls were healthy people, whereas mutant controls were MAS patient-derived *GNAS* mutated tissues. Informed consent for genetic studies was obtained from all subjects involved in the study.

DNA samples were extracted from peripheral blood in EDTA collection tubes (Flexigene DNA kit, Qiagen, Germany) and fresh or FFPE tissue samples (GentraPuregene tissue kit, Qiagen, Germany), according to the manufacturer’s instructions.

### Techniques for the Detection of GNAS MAS-Related Genetic Variants

To investigate the presence of heterozygous gain-of-function *GNAS* variants c.604C > T/p.Arg202Cys and c.605G > A/p.Arg202His, commonly reported in the literature as Arg201 (in accordance with the guidelines recommended by the Human Genome Variation Society (HGVS), http://www.hgvs.org/mutnomen/, for a uniform and an unequivocal description of sequence variants in DNA and protein sequences, the nucleotide and protein numbering used in the present article was based on the Locus Reference Genomic (LRG) sequence, https://www.lrg-sequence.org/, covering the *GNAS* transcript NM_001077488.2; the *GNAS* mutations database available at http://databases.lovd.nl/shared/genes/GNAS) associated with the MAS/FD phenotype, different experimental techniques were used: Sanger sequencing, allele-specific PCR (AS-PCR), allele-specific PCR-based TaqMan genotyping with co-amplification at lower denaturation temperature (COLD-MAMA PCR), and dPCR.

Detection limits (LOD) of each method, defined as the lowest concentration that can be reliably distinguished from healthy controls, are a consequence of assay specificity, sample quality/handling, and PCR inhibitors. For the empirical determination of detection limits, we applied the strategy of analyzing no DNA template controls (NTCs, contamination monitors), wild-type negative controls (WTCs, false-positive monitors), and a serial dilution of mutant positive controls in a background of wild-type DNA (MTCs, positive controls for thresholding and making limit of detection calls).

The *GNAS* exon 8 was amplified using the specific couple of primers 5’-ACTCTGAGCCCTCTTTCCAA-3’ and 5’-GGTAACAGTTGGCTTACTGG-3’ (thermal conditions: 94°C/5’, 40 cycles at 94°C/30”, 58°C/30”, and 72°C/30”, 72°C/5’). Sanger sequencing was performed using the AmpliTaqBigDye Terminator kit and the 3110xl Genetic Analyzer (Applied Biosystems, Foster City, CA, USA).

Amplicons subjected to sequencing were also used as template for AS-PCRs, visualized and analyzed on 3% agarose gels, that was shown to be able to discriminate wild-type and mutant alleles using sequence-specific primers (c.604C 5′-GATTCCAGAAGTCAGGACACG-3′, c.604T 5′-GATTCCAGAAGTCAGGACACA-3′, c. 605G 5′-GATTCCAGAAGTCAGGACAC-3′, and c.605° 5′-GATTCCAGAAGTCAGGACAT-3′) under stringent thermal conditions (94°C/3’, 40 cycles at 94°C/15”, 67°C/15”, and 72°C/15”, 72°C/3’).

The GNAS locus fragment bearing MAS/FD -associated variants was investigated by COLD-MAMA PCR, as previously described (de Sanctis et al., 2017).

**Figure 1:**
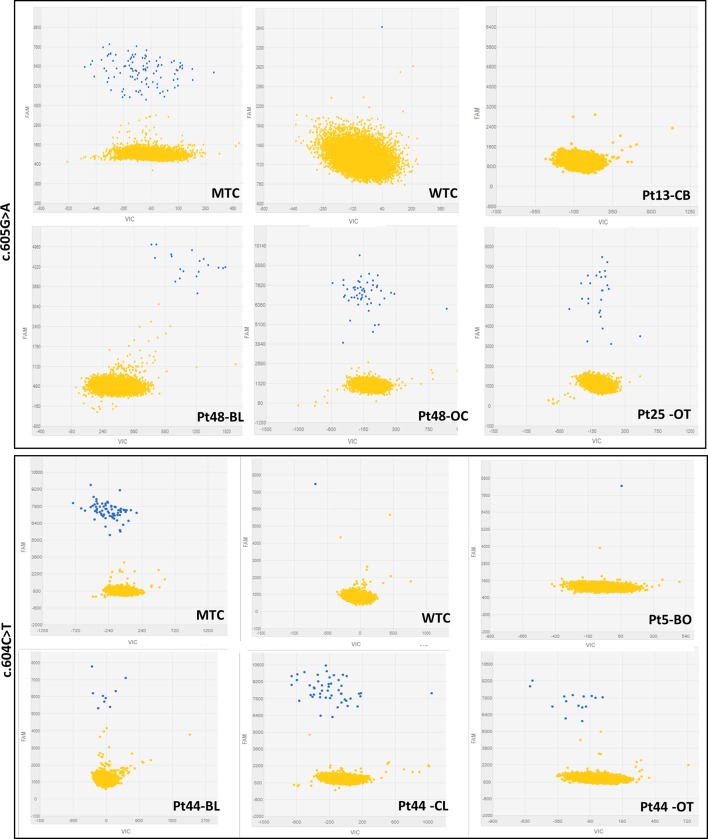
Representative 2-D scatterplots obtained from the raw imaging data analysis of rare mutation detection analysis using the QuantStudio™ 3D AnalysisSuite™ Software (upper panel: GNAS_27895_mu assay; lower panel: GNAS_27877_mu assay). Blue dots represent the FAM™ reporter dye signal that allows to identify and count mutant alleles present in the nanoscale-sized reaction wells of the chip. Yellow dots are the assay control signal and represent the absence of FAM™ signal in part of the nanoscale-sized reaction wells of the chip.

The dPCR was performed with the QuantStudio™ 3D Digital PCR System platform (all products by ThermoFisher Scientific, Carlsbad, CA, USA) and predesigned FAM-labelled TaqMan^®^ Mutation Detection Assays, GNAS_27895_mu (c.605G > A), and GNAS_27887_mu (c.604C > T). Briefly, 1 μl of 5 ng/μl DNA (A260/280 ratios between 1.7 and 1.9) diluted in 14 μl of reaction mixture (8 μl QuantStudio™ 3D Digital PCR Master Mix, 1.6 μl TaqMan^®^ Mutation Detection Assay, and 4.4 μl nuclease-free water) were loaded into QuantStudio™ 3D Digital PCR 20K Chips and amplified (thermal conditions: 1 cycle at 96°C/10’, 40 cycles at 66°C/1’, and 98°C/45’, 1 cycle at 60°C/1’). Fluorescence on chips was revealed by the QuantStudio™ 3D Instrument, and collected raw data were analyzed by the QuantStudio™ 3D AnalysisSuite™ Software. The software assesses reliable data and displays quality indicators, based upon loading, signal, and noise characteristics, for each chip. This quality control is based on the number of partitions that exceed the total number of wells filled correctly, and to get a precise quantification, we settled a threshold of 10,000 data points with a manually fixed quality threshold over 0.6. Performance experiments conducted for both assays determined the appropriate fluorescence thresholds in FAM relative fluorescence units (RFU) to discriminate between positive calls (blue dots) and no target control calls (yellow dots) (**Figure 1**): GNAS_27,895_mu RFU > 3,000 and GNAS_27,887_mu RFU > 5,000. The output data are reported as copies/μl (cpm) detected on the chip by the instrument that represents the number of observed mutant alleles in the reagent mixture plus DNA. The absolute quantification of mutated alleles can be deduced from the cpm by applying the following formula: cpm X 0.0033 ng (mass of the human genome) X 3.34 (dilution factor for 15 μl of sample plus reaction mixture) = ng/μl.

The specificity and the sensitivity of the dPCR technique were assessed testing NTCs, WTCs, and MTCs, previously identified by Sanger sequencing, and a calibrator curve, in triplicate, of serially diluted mutated samples (representing 100% MTCs), ranging from 50% to 3% and from 25% to 1.5% of c.604 or c.605 heterozygously mutated samples, respectively, representing the relative mutation abundance (RMA). Cutoff values, or rather copies of mutated DNA per 1 μl of the tested sample, cpm, to diagnose the presence of a mutation and the RMA in each patient’s sample were then inferred by comparing observed cpm values with those detected by the calibrator curve.

## Results

The bulk of experiments performed allowed to obtain a molecular diagnosis in MAS/FD subjects that were still missing because of the low detection rate of the Sanger sequencing, thanks to the setup of the dPCR, a highly sensitive and specific technique to detect low-abundance somatic single-nucleotide *GNAS* alterations. To validate results produced with the dPCR, we also compared this novel approach with additional available methods to search for somatic genetic alterations, the AS-PCR and the COLD-MAMA PCR.

**Figure 2:**
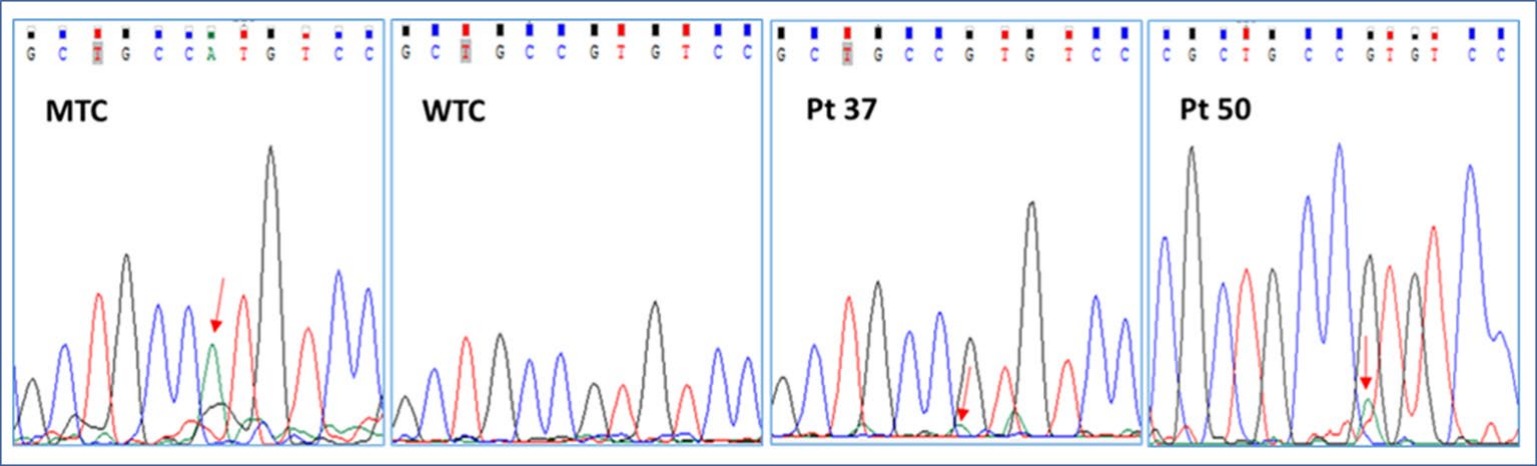
Representative electropherograms of the GNAS c.605G > A mutation screening by Sanger sequencing of a mutant control (MTC), a wild-type control (WTC), and selected patients (pt37-BO-RJ and pt50-OC). Red arrows highlight the position of the c.605A nucleotide.

We started our investigation with a careful analysis of Sanger electropherograms obtained by both calibrator curves and MAS/FD patients, showing that the mutated allele was clearly detectable only in case of samples containing elevated amounts of mutated DNA (LOD > 0.1 ng/μl) but only conceivable for lower RMAs (**Figure 2**; **Table 2**). As a matter of fact, the high background noise was a side effect that made it impossible to discriminate real calls from nonspecific signals, thus leading to the failure to establish patients’ genotype conclusively.

In particular, the Sanger sequencing detected somatic mutations only in 6 of 79 tested DNA samples (5/15 BO, 33.3%; 0/16 CB/FB, 0%; 1/11 OC/OCL/OT, 9%; 0/37 BL, 0%) belonging to 4 of 54 different patients (7.4% detection rate) (**Supplementary Table 2**; **Tables 1** and **2** panel A). No patient affected by the c.604C > T variant was found. On the other hand, four patients (pt2-BO, pt3-BO, pt37-BO-LJ+M+RJ, and pt46-OC) obtained a positive diagnosis for the c.605G > A variant. The pathogenetic variant was found by Sanger only in DNA samples extracted from affected biopsies. These data further highlighted the technical limitations in performing a rare mutation investigation by Sanger in DNA samples from whole blood.

**Table 1:** Table resuming molecular data of the studied cohort of MAS patients. Cpm, copies/µl. Abs quant, absolute quantification.

		c.604C>T								c.605G>A							
PT ID	TISSUE	SANGER	AS-PCR	dPCR	dPCR cpm	dPCR absquant (ng/µl)	dPCR RMA	COLD-MAMA PCR	COLD-MAMA RMA	SANGER	AS-PCR	dPCR	dPCR cpm	dPCR absquant (ng/µl)	dPCR RMA	COLD-MAMA PCR	COLD-MAMA RMA
**1**	**BO**	WT	WT	WT	0.181	0.00				WT	WT	**MUT**	**0.892**	**0.01**	**>6**		
**2**	**BO**	WT	WT	WT	0.000	0.00				**MUT**	**MUT**	**MUT**	**8.712**	**0.10**	**>50**		
**3**	**BO**	WT	WT	WT	0.095	0.00				**MUT**	**MUT**	**MUT**	**17.626**	**0.19**	**100**		
**4**	**BO**	WT	WT	WT	0.000	0.00				WT	WT	WT	0.169	0.00			
**5**	**BO**	WT	nd	WT	0.000	0.00		nd		WT	nd	**MUT**	**6.594**	**0.07**	**50**	nd	
**6**	**BO**	WT	WT	WT	0.000	0.00		WT		WT	**MUT**	**MUT**	**3.604**	**0.04**	**25**	**MUT**	**3**
**7**	**BO**	WT	WT	WT	0.000	0.00				WT	WT	WT	0.000	0.00			
**8**	**BO**	WT	WT	WT	0.086	0.00				WT	WT	WT	0.353	0.00			
**12**	**BO**	WT	nd	WT	0.260	0.00		nd		WT	nd	**MUT**	**3.196**	**0.04**	**25**	nd	
**9**	**BO**	WT	nd	WT	0.255	0.00		nd		WT	nd	WT	0.084	0.00		nd	
**37**	**BL**	WT	WT	WT	0.166	0.00		WT		WT	WT	**MUT**	**0.622**	**0.01**	**>6**	**MUT**	**6**
	**BO-LJ**	WT	WT	WT	0.195	0.00		WT		**MUT**	**MUT**	**MUT**	**32.654**	**0.36**	**100**	**MUT**	**>40**
	**BO-M**	WT	nd	WT	0.000	0.00		nd		**MUT**	nd	**MUT**	**22.108**	**0.24**	**100**	nd	
	**BO-RJ**	WT	WT	WT	0.169	0.00		WT		WT	**MUT**	**MUT**	**2.121**	**0.02**	**<25**	**MUT**	**3**
	**BO-S**	WT	nd	WT	0.188	0.00		nd		**MUT**	nd	**MUT**	**22.904**	**0.25**	**100**	nd	
**45**	**BL**	WT	WT	WT	0.087	0.00				WT	WT	WT	0.312	0.00			
	**BO**	WT	WT	WT	0.092	0.00				WT	WT	WT	0.105	0.00			
**10**	**CB**	WT	WT	WT	0.152	0.00				WT	WT	WT	0.343	0.00			
**11**	**CB**	WT	WT	WT	0.237	0.00				WT	WT	WT	0.436	0.00			
**13**	**CB**	WT	WT	WT	0.105	0.00				WT	WT	WT	0.100	0.00			
**14**	**CB**	WT	WT	WT	0.000	0.00				WT	WT	WT	0.185	0.00			
**38**	**BL**	WT	WT	WT	0.000	0.00				WT	WT	WT	0.178	0.00			
	**CB-WT**	WT	WT	WT	0.183	0.00				WT		WT	0.233	0.00			
	**CB-YT**	WT	WT	WT	0.105	0.00				WT		WT	0.416	0.00			
	**CB-GT**	WT	WT	WT	0.173	0.00				WT		WT	0.191	0.00			
**39**	**BL**	WT	WT	WT	0.319	0.00				WT	WT	**MUT**	**0.509**	**0.01**	**6**		
	**CB**	WT	WT	WT	0.000	0.00				WT	WT	**MUT**	**1.251**	**0.01**	**<12.5**		
**52**	**BL**	WT	WT	WT	0.112	0.00		nd		WT	**MUT**	**MUT**	**0.598**	**0.01**	**6**	nd	
	**CB**	WT	WT	WT	0.000	0.00		nd		WT	**MUT**	**MUT**	**6.208**	**0.07**	**50**	nd	
**40**	**BL**	WT	WT	WT	0.000	0.00		WT		WT	WT	WT	0.096	0.00		WT	
	**CB**	WT	WT	WT	0.320	0.00		nd		WT	WT	WT	0.139	0.00		WT	
**41**	**BL**	WT	WT	**MUT**	**2.249**	**0.02**	**<12.5**	**MUT**	**<2.5**	WT	WT	WT	0.254	0.00		WT	
	**CB-GT**	WT	WT	WT	0.104	0.00				WT	WT	WT	0.078	0.00			
	**CB-WT**	WT	WT	WT	0.000	0.00		nd		WT	WT	WT	0.336	0.00		nd	
	**CB-YT**	WT	WT	WT	0.000	0.00				WT	WT	WT	0.180	0.00			
**42**	**BL**	WT	WT	WT	0.223	0.00		WT		WT	WT	WT	0.083	0.00		WT	
	**FB**	WT	WT	WT	0.265	0.00		WT		WT	WT	WT	0.190	0.00		WT	
**43**	**BL**	WT	WT	WT	0.188	0.00				WT	WT	**MUT**	**0.873**	**0.01**	**>6**		
	**CB**	WT	WT	WT	0.000	0.00				WT	WT	WT	0.089	0.00			
	**FB**	WT	WT	WT	0.000	0.00				WT	WT	**MUT**	**1.633**	**0.02**	**>12.5**		
**35**	**OT**	WT	nd	WT	0.000	0.00				WT	nd	WT	0.244	0.00			
**44**	**BL**	WT	WT	**MUT**	**0.777**	**0.01**	<3	WT		WT	WT	WT	0.293	0.00		WT	
	**OCL**	WT	WT	**MUT**	**4.995**	**0.06**	**<25**	**MUT**	**<2.5**	WT	WT	WT	0.256	0.00		WT	
	**OT**	WT	WT	**MUT**	**1.485**	**0.02**	>6	WT		WT	WT	WT	0.184	0.00		WT	
**46**	**OC**	WT	WT	WT	0.083	0.00		WT		**MUT**	**MUT**	**MUT**	**2.969**	**0.03**	**<25**	**MUT**	**10**
	**OT**	WT	WT	WT	0.182	0.00		WT		WT	WT	**MUT**	**0.520**	**0.01**	**6**	**MUT**	**<0.5**
**47**	**BL**	WT	WT	WT	0.000	0.00		WT		WT	WT	WT	0.299	0.00		WT	
	**OC**	WT	WT	WT	0.102	0.00		WT		WT	WT	**MUT**	**1.135**	**0.01**	**<12.5**	WT	
	**OT**	WT	WT	WT	0.084	0.00		WT		WT	WT	**MUT**	**1.164**	**0.01**	**<12.5**	**MUT**	**3**
**48**	**BL**	WT	WT	WT	0.334	0.00		WT		WT	**MUT**	**MUT**	**5.347**	**0.06**	**<50**	**MUT**	**16**
	**OC**	WT	WT	WT	0.000	0.00		WT		WT	**MUT**	**MUT**	**10.169**	**0.11**	**>50**	**MUT**	**>40**
**49**	**BL**	WT	WT	WT	0.101	0.00		WT		WT	WT	**MUT**	**0.465**	**0.01**	**3**	WT	
	**OC**	WT	WT	WT	0.192	0.00		WT		WT	WT	**MUT**	**1.578**	**0.02**	**>12.5**	**MUT**	**26**
**50**	**OC**	WT	WT	WT	0.412	0.00		WT		WT	**MUT**	**MUT**	**3.312**	**0.04**	**25**	**MUT**	**<0.5**
	**OCL**	WT	WT	WT	0.205	0.00		WT		WT	**MUT**	**MUT**	**7.275**	**0.08**	**>50**	**MUT**	**2.5**
**15**	**BL**	WT	WT	WT	0.000	0.00				WT	WT	WT	0.191	0.00			
**16**	**BL**	WT	WT	WT	0.115	0.00				WT	WT	WT	0.000	0.00			
**17**	**BL**	WT	WT	WT	0.154	0.00				WT	WT	WT	0.264	0.00			
**18**	**BL**	WT	WT	WT	0.101	0.00				WT	WT	WT	0.000	0.00			
**19**	**BL**	WT	WT	WT	0.117	0.00		WT		WT	WT	**MUT**	**0.815**	**0.01**	**>6**	WT	
**20**	**BL**	WT	WT	WT	0.097	0.00				WT	WT	WT	0.119	0.00			
**21**	**BL**	WT	WT	WT	0.089	0.00				WT	WT	WT	0.308	0.00			
**22**	**BL**	WT	WT	WT	0.000	0.00				WT	WT	**MUT**	**0.644**	**0.01**	**6**		
**23**	**BL**	WT	WT	WT	0.000	0.00				WT	WT	WT	0.219	0.00			
**24**	**BL**	WT	WT	WT	0.000	0.00				WT	WT	WT	0.000	0.00			
**25**	**BL**	WT	WT	WT	0.000	0.00		nd		WT	WT	**MUT**	**2.252**	**0.02**	**>12.5**	nd	
**26**	**BL**	WT	WT	WT	0.026	0.00				WT	WT	WT	0.000	0.00			
**27**	**BL**	WT	WT	WT	0.000	0.00				WT	WT	WT	0.000	0.00			
**28**	**BL**	WT	WT	WT	0.000	0.00		nd		WT	**MUT**	**MUT**	**2.170**	**0.02**	**>12.5**	nd	
**29**	**BL**	WT	WT	WT	0.000	0.00				WT	WT	WT	0.285	0.00			
**31**	**BL**	WT	WT	WT	0.095	0.00		WT		WT	WT	WT	0.383	0.00		WT	
**32**	**BL**	WT	WT	WT	0.277	0.00		nd		WT	WT	WT	0.000	0.00		WT	
**33**	**BL**	WT	WT	WT	0.000	0.00				WT	WT	WT	0.190	0.00			
**34**	**BL**	WT	WT	WT	0.000	0.00				WT	WT	WT	0.000	0.00			
**51**	**BL**	WT	nd	WT	0.000	0.00		nd		WT	nd	**MUT**	**1.216**	**0.01**	**12.5**	nd	
**53**	**BL**	WT	nd	WT	0.258	0.00		nd		WT	nd	**MUT**	**1.043**	**0.01**	**<12.5**	nd	
**54**	**BL**	WT	nd	WT	0.000	0.00		nd		WT	nd	WT	0.246	0.00		nd	
**30**	**BL**	WT	nd	WT	0.000	0.00		nd		WT	nd	WT	0.171	0.00		nd	
**36**	**BL**	WT	WT	WT	0.150	0.00				WT	WT	WT	0.202	0.00			

RMA, relative mutation amount; BO, bone; BL, blood; LJ, left jaw; M, maxilla; RJ, right jaw; S, symphysis; CB, cutaneous biopsy; FB, fibroblast; OCL, ovarian cyst liquid; OC, ovarian cyst; OT, ovarian tissue; nd, not determined; WT, wild-type. Bold data highlight results obtained in mutated patients.

**Table 2:** Tables resuming the detection rate of MAS-related c.604T and c.605A *GNAS* activating variants.

	Sanger	AS-PCR	COLD-MAMA PCR	dPCR
A				
**BO**	5/15; **33.3%**	5/10; **50%**	3/3; **100%**	10/15; **66.6%**
**CB/FB**	0/16; **0%**	1/16; **6.25%**	0/2; **0%**	3/16; **18.75%**
**OC/OCL/OT**	1/11; **9%**	4/10; **40%**	7/10; **70%**	10/11; **90.9%**
**BL**	0/37; **0%**	3/33; **9.1%**	2/11; **18.1%**	14/37; **37.8%**
**Patients (overall DR)**	4/54; **7.4%**	9/46; **19.6%**	9/14; **64.3%**	23/54; **42.6%**
**B**				
**BO**	0/3; **0%**	3/3; **100%**	3/3; **100%**	3/3; **100%**
**CB/FB**	0/2; **0%**	0/2; **0%**	0/2; **0%**	0/2; **0%**
**OC/OCL/OT**	1/10; **10%**	4/10; **40%**	7/10; **70%**	10/10; **100%**
**BL**	0/11; **0%**	1/11; **9%**	2/11; **18.1%**	6/11; **54.5%**
**Patients (overall DR)**	2/14; **14.3%**	5/14; **35.7%**	9/14; **64.3%**	10/14; **71.4%**

BO, bone; CB/FB, cutaneous biopsy/fibroblasts; OC/OCL/OT, ovarian cyst/ovarian cyst liquid/ovarian tissue; BL, blood; DR, detection rate.

Panel A reports all patients whereas panel B reports only patients tested by all of the four available techniques. Percentages values are highlighted in bold.

**Figure 3:**
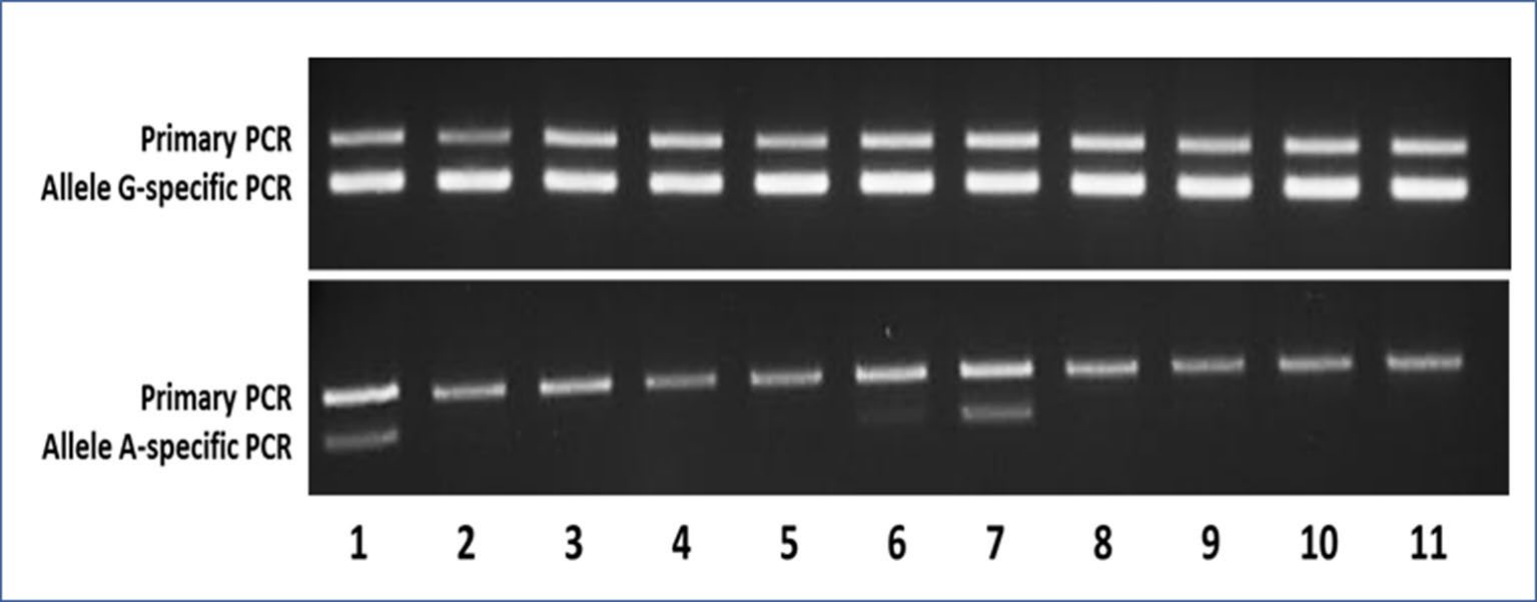
Representative agarose gels of the AS-PCR for the detection of the c.605G > A variant showing both the aspecific primary PCR on top and the allele-specific nested PCR below amplicon bands. In the upper gel, as expected, the G-specific amplicon was appreciable for each sample, whereas in the lower gel, the A-specific amplicon was detectable only in samples bearing the mutant A allele. Samples 1–9 were MAS patients (1–2 = pt46-OC+OT; 3–5 = pt47-BL+OC+OT; 6–7 = pt48-BL+OT; 8–9 = pt49-BL+OT), whereas samples 10 and 11 were WTCs.

The use of allele-specific primers allowed to slightly improve the detection threshold of MAS/FD-related variants (LOD > 0.03 ng/μl) as it discovered 13 samples/66 with the c.605A variant, belonging to 7 of 46 different patients: pt2-BO, pt3-BO, pt28-BL, pt37- BO-LJ (left jaw)+RJ (right jaw), pt52-BL+CB, pt46-OC, and pt48-BL+OC), but the sensitivity of the AS-PCR (19.6% detection rate) was still too low to perform an accurate molecular diagnosis of MAS in most patients (**Supplementary Table 2**; **Tables 1** and **2** panel A; **Figure 3**). All Sanger-positive samples were confirmed, and three novel positive patients were found. It is noteworthy that, contrary to Sanger, this method allowed to discover a mutation even in three blood samples (5/10 BO, 40%; 1/16 CB/FB, 6.25%; 4/10 OC/OCL/OT, 40%; 3/33 BL, 9.1%) (**Table 2** panel A). No false-positive/negative results were obtained, and the interindividual and intraindividual, or rather different tissues from the same patient, differences in RMAs were the same as observed by dPCR, thus excluding an artifact or carryover contamination.

Forty samples from 24 patients were prepared for the COLD-MAMA PCR but, because of the insufficient amount of available DNA, only 24 samples from 14 patients for the c.604C > T and 26 samples from 14 patients for the c.605G > A were successfully analyzed, demonstrating the presence of the T-mutated allele in 2/24 samples (pt41-BL and pt44-OCL) and of the A-mutated allele in 12/26 samples (pt6-BO, pt37-BL+BO-LJ+RJ, pt46-OC+OT, pt47-OT, pt48BL+OC, pt49-OC, pt50-OC+OCL), showing the presence of a MAS/FD causative genetic variant in 9 of 14 patients with an overall detection rate of 64.3% (3/3 BO, 100%; 0/2 CB/FB, 0%; 7/10 OC/OCL/OT, 70%; 2/11 BL, 18.1%) and a LOD > 0.02 ng/μl (**Supplementary Table 2**; **Tables 1** and **2** panel A).

The first phase of dPCR data analysis, performed using the QuantStudio™ 3D AnalysisSuite™ Software, relied on manual examination and thresholding 2-D scatterplots from control samples (NTCs, WTCs, and MTCs) (**Figure 1**). Neither false-positive nor false-negative was observed among tested WTCs (c.604T n = 9, c.605A n = 13) and MTCs (c.604T n = 3, c.605A n = 6), confirming the good performance of both assays. Thus, the dPCR allowed to discriminate all tested MTCs from WTCs.

**Figure 4:**
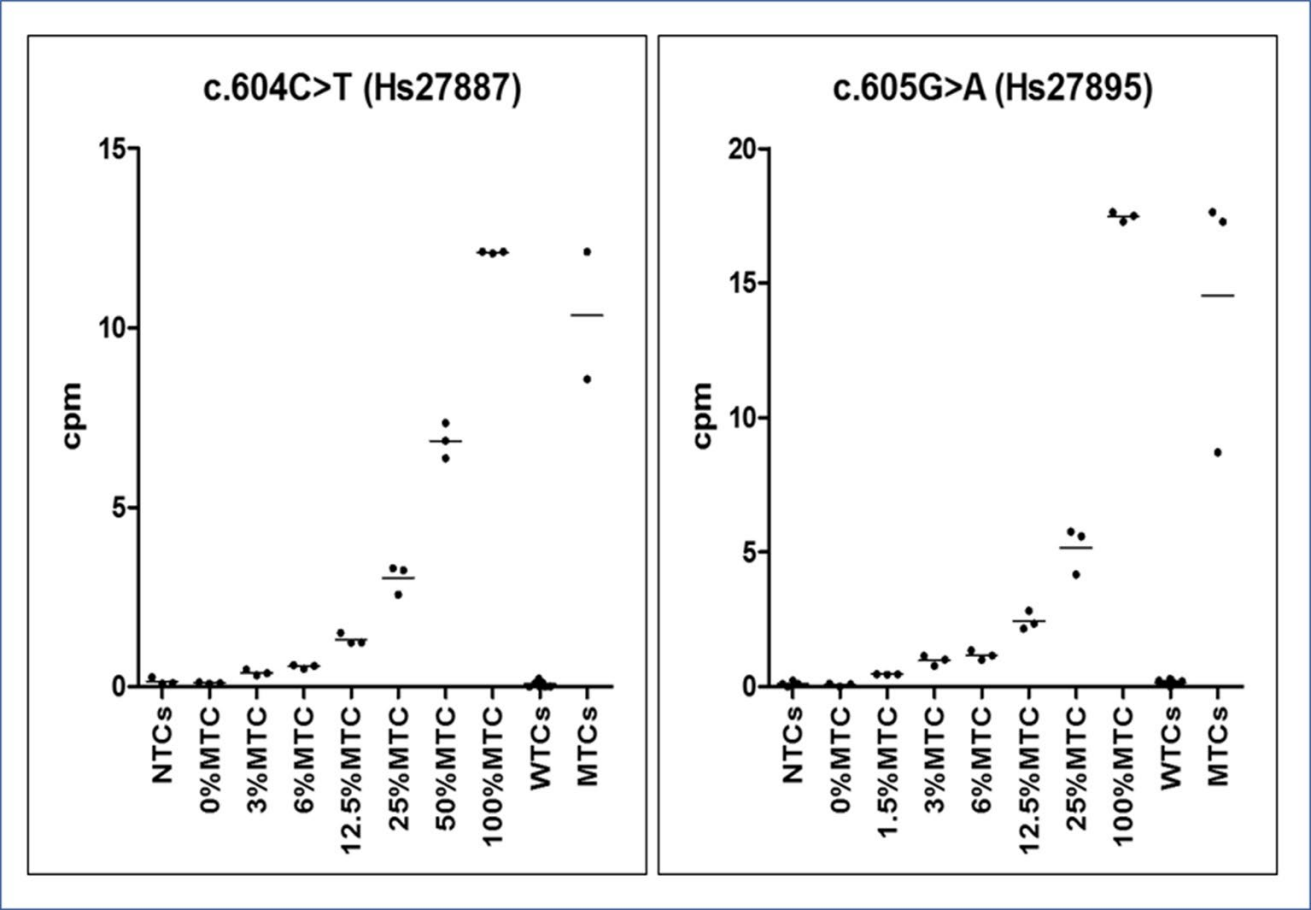
Graphs of dPCR cpm calibrator curves. On the Y-axis, the copies per µl (cpm) of mutated allele are reported, whereas on the X-axis, the sample type (no template ctrls - NTCs. wild-type template ctrls - WTCs and mutated template ctrls - MTCs). Observed reference cpm (mean of triplicates ± SD/% RMAs) were 0.102 ± 0.017/0%, 0.391 ± 0.074/3%, 0.563 ± 0.052/6%, 1. 321 ± 0.157/12.5%, 3.035 ± 0.411/25%, 6.855 ± 0.494/50%, and 12.11 ± 0.023/100% for the c.604T variant and 0.068 ± 0.06/0%, 0.464 ± 0.012/1.5%, 0.976 ± 0.189/3%, 1.171 ± 0.181/6%, 2.44 ± 0.346/12.5%, 5.17 ± 0.873/25%, and 17.47 ± 0.177/100% for the c.605A variant. All points of the serial dilution resulted significantly different (CI 95%) from the 0%MTC.

From calibrator curves of serially diluted MTCs, mimicking various RMAs, we determined the LOD of 0.01 ng/μl, reference cpm values were used as cutoffs for inferring the presence of a *GNAS* somatic mutation in patients and the RMA in each patient’s sample (**Figure 4**). According to the *t* test (95% CI), all points of the serial curve resulted differently from 0% RMA samples, and statistically significant cutoffs to detect the positiveness of a sample were 0.391 ± 0.074 (P = 0.0028) for the c.604T and 0.464 ± 0.012 (P = 0.0004) for the c.605A alleles. Note that TaqMan^®^ Mutation Detection Assays showed distinct lower detection thresholds, 3% RMA for the GNAS_27887_mu (p.Arg202Cys) and 1.5% RMA for the GNAS_27895_mu (p.Arg202His), but the best fitting explanation for this slightly contrasting performance was the use of DNA serial dilutions prepared from MTCs with a different starting concentration and, consequently, a different RMA (0.13 ng/μl and RMA 12.11 ± 0.023 and 0.19 ng/μl and RMA 17.47 ± 0.177, respectively) rather than a different conduct of assays or a setup failure.

During the analysis of patient samples, the short way to call a positive sample was by visualizing the 2-D plot and comparing cutoff cpm values, considering nonoverlapping error bars, between WTCs and unknown samples, whereas a *t*-test was used to determine the correct RMA subcluster attribution of positive samples.

The dPCR allowed identification of the mutation in 36/79 samples (10/15 BO, 66.6%; 3/16 CB/FB, 18.75%; 10/11 OC/OCL/OT, 90.9%; 14/37 BL, 37.8%) in 23/54 patients (42.6% detection rate), of whom 2 patients (pt41-BL and pt44-BL+OCL+OT) carried the c.604T variant and 21 patients (pt1-BO, pt2-BO, pt3-BO, pt5-BO, pt6-BO, pt12-BO, pt19-BL, pt22-BL, pt25-BL, pt28-BL, pt51-BL, pt53-BL, pt37-BL+BO, pt39-BL+CB, pt52-BL+CB, pt43-BL+FB, pt46-OC+OT, pt47-OC+OT, pt48-BL+OC, pt49-BL+OC, and pt50-OC+OCL) the c.605A variant (**Supplementary Table 2**; **Tables 1** and **2** panel A). In particular and importantly, the technique was able to identify a mutation in 37.8% of peripheral blood samples.

The comparison of data obtained in seven mutated patients from whom we collected both blood and affected tissues (pt37, pt39, pt43, pt 44, pt48, pt49, and pt52) ensured the ability of dPCR to detect MAS/FD-associated alleles in gDNA extracted from blood samples with an RMA sufficient to be ascertained by the system.

Curiously, patient 41’s cutaneous biopsy resulted negative for alterations, but we found the mutation in two independently collected blood samples by both dPCR and COLD-MAMA PCR. In patient 43, we found the mutated allele both in the blood and in cultured fibroblasts but not in the DNA extracted from the cutaneous biopsy (**Supplementary Table 2**; **Table 1**).

## Discussion

The work presented in this paper aimed at setting up a novel technology, the dPCR, to perform the molecular diagnosis of MAS/FD by the identification of rare somatic activating variants of the *GNAS* gene. The detection of low-level somatic variants in MAS/FD patients is a challenge for the laboratory because it relies on the discrimination between two highly similar sequences, of which the wild-type one is significantly more abundant in the sample. Molecular procedures currently used in our laboratory, such as Sanger sequencing and AS-PCR, do not reach the needed sensibility, thus failing to detect rare mutations. For this reason, most MAS/FD patients often lack a molecular confirmation of their disease.

Up to now, scientists developed several methods to overcome technical limitations associated with performing a successful genetic diagnosis of MAS/FD (i.e., enrichment methods with subsequent restriction enzyme digestion, RFLP, or with peptide nucleic acid probes, PNA, and next-generation sequencing, NGS, with or without PNA) (Schwindinger et al., 1992; Liang et al., 2011; Hannon et al., 2003). Selective enrichment methods reached good levels in low-abundance variant identification, but their execution is particularly cumbersome and time-consuming, as the requirement of a large number of PCR cycles for the selective amplification of the mutated allele leads to an elevated risk to create PCR artifacts and cross-contamination. An opportunity to overcome these limitations would be an approach such as the deep-sequencing NGS (depth of sequence coverage approximately 800X) with molecular bar code technique, although it is more expensive and still far from clinical settings.

During the setup phase, the dPCR allowed to discriminate all tested WTCs and MTCs. It showed an extremely high sensitivity, being able to detect even few copies of mutated alleles in DNA samples extracted from different tissues, including blood samples.

To validate the dPCR approach, we analyzed our samples also by Sanger sequencing, AS-PCR, and COLD-MAMA PCR. Positive results obtained by Sanger, AS-PCR, and COLD-MAMA PCR were in full agreement with those by dPCR.

As expected, the direct sequencing of *GNAS* exon 8, confirmed to be the less sensitive method, was able to reach in our cohort only a 7.4% detection rate, which means in only 4 of the 54 analyzed patients. Mutated alleles were found exclusively in a small subset of biopsies, mainly from bone, whereas no mutation was detected in blood samples. The AS-PCR allowed to slightly improve the overall detection rate, raising it to 19.6% (7 of 46 analyzed patients) and to identify three positive patients directly from the blood-extracted DNA, two of them confirmed in the affected tissue as well. The COLD-MAMA PCR reached an overall 64.3% detection rate (9 of 14 analyzed patients) but needed, as the AS-PCR, a large amount of starting material with respect to the dPCR that limited the opportunity to test all of the patients of our cohort and to compare final diagnoses. This method identified almost all mutated tissue samples but in only 2 of the 11 blood-extracted samples (18.1% detection rate). Finally, the dPCR obtained an overall 42.6% detection rate by finding 23 mutated patients of the 54 tested ones. In particular, this latter technique discovered low amounts of mutated alleles in 14/37 blood samples, with a detection rate of 37.8%. (**Table 2** panel A). We consider noteworthy to show the success rate of the dPCR because of the fact that this technique allowed to find false-negative (FN) patients by the other methods (19 Sanger FN, 10 AS-PCR FN, and 1 COLD-MAMA PCR FN).

In the present work, we investigated the two most common mutations affecting the amino acid residue R202 (previously reported as R201), the p.R202H and p.R202C, that account for most of diagnosed MAS-associated genetic defects. In ultra-rare cases, additional substitutions have been reported at position 202, p.R202S/L/G, and at position 228, p.Q228L/R/K/H (Candeliere et al., 1997; Riminucci et al., 1999; Lietman et al., 2005; Idowu et al., 2007). The existance of such exceptional variants and the possibility to discover novel activating GNAS defects might explain for the portion of MAS patients still missing a confirmative molecular diagnosis that includes both true- and false-negative patients.

To compare the efficiency of the different analytical methods in detecting mutated MAS/FD patients, we analyzed separately the cluster of samples and patients investigated by all four of them and determined the detection rate for patients and by single sample type. This analysis showed an increase in sensitivity leading to the improvement of the detection rate in the considered subcohort of 14 cases (14.3% for Sanger, 35.7% AS-PCR, 64.3% for COLD-MAMA PCR, and 71.4% for dPCR), indicating dPCR as the most performing approach. When we considered the testing of specific tissues, we observed that all techniques, but Sanger, found the mutated allele in bone samples, whereas in ovarian tissues and blood, we noticed a progressive improvement in finding rare variants from the lowest by Sanger to the highest by dPCR (**Table 2** panel B). Moreover, the dPCR approach showed to be less susceptible to poor sample quality and to the presence of inhibitors because it demonstrated to be able to successfully analyze all of the cohort of samples, including very small amounts of DNA after long-term storage and FFPE-extracted DNAs. In particular, the other methods failed in some cases, which were reported as “not determined” in **Table 2**, because of the reduced amount or the poor quality of material available for testing.

Detecting mutated alleles from blood sample was the final and more important goal of our study because we wanted to obtain an analytical method for the noninvasive testing of MAS/FD patients, overcoming the need for invasive procedures like biopsies. Checking the detection rates in the subcohort of DNAs from peripheral blood, dPCR achieved the highest performance with respect to the other techniques (54.5% dPCR versus 18.1% COLD-MAMA PCR versus 9% AS-PCR versus 0% Sanger) (**Table 2** panel B). In seven patients, results were cross-confirmed by the genotype found in tissue specimens (pt37, pt39, pt43, pt44, pt48, pt49, and pt52) (**Supplementary Table 2**; **Table 1**). We agree with Narumi and colleagues’ (2013) observation that the relative mutation abundance in blood is strikingly variable, it does not reflect distribution and extension of affected lesions, and it does not correlate with disease severity and progression, as already observed in other congenital syndromes caused by somatic genetic variants (i.e., Proteus and megalencephaly syndromes) (Lindhurst et al., 2011; Rivière et al., 2012; Narumi et al., 2013).

Our calibrator curves, being derived from a serial dilution of mutated patients and not cloned DNA, allowed a relative rather than an absolute quantification of the RMA in samples, and we used them to define reference cutoff cpm values to discriminate between wild-type and mutated samples. We do not consider this fact as a limit of the present study that was aimed at improving the number of successful molecular diagnosis of MAS/FD because the absolute RMA showed no real utility from a clinical point of view and it did not affect the dPCR performance because this method carries out an absolute quantitation of nucleic acid samples based on PCR amplification and absolute count of single template molecules.

Data from previously published papers showed that the dPCR is an extremely competitive method. For example, Lumbroso and colleagues (2004) studied a very big series of MAS patients and determined the frequency of observed mutated patients in several different tissues by RFLP analysis. The dPCR reached a better performance in the blood (37.8% dPCR versus 21% RFLP) and ovarian tissues (90.9% by dPCR versus 65% by RFLP) (Lumbroso et al., 2004).

If we consider ours and Lumbroso’s data on tissue samples, 10 of 15 (66.6%) and 9 of 11 (82%) bone biopsies, 2 of 14 (14.3%) and 3 of 11 (27%) cutaneous biopsies, 1 of 2 (50%) and 0 fibroblasts, 5 of 5 (100%) and 3 of 8 (38%) ovarian cysts, 2 of 2 (100%) and 13 of 19 (68%) ovarian cyst liquids and 3 of 4 (75%) and 10 of 13 (77%) ovarian tissues were positive by RFLPs and dPCR, respectively (Narumi et al., 2013). Even if ours and Lumbroso’s are different techniques, this comparison allowed to appreciate the different detection rates of mutated alleles related to the specific tested tissue and to confirm, in accordance with Vasilev and colleagues (2014), that tissues atypically/rarely involved in MAS may have the *GNAS* mutation in mosaic distribution (Narumi et al., 2013; Vasilev et al., 2014).

Bone was the best tissue to identify MAS/FD rare variants, which were found both in patients showing isolated FD and the classic MAS triad. In both our samples of ovarian cystic fluid, we identified the mutated allele, confirming its usefulness also in case of isolated precocious puberty. The systematic study of the ovarian cystic fluid would be advisable for its major implications for both disease progression and treatment.

Our data are also in line with the observation that some samples may remain apparently negative because the biopsy missed loci where mutated cells are confined, and that specimens like skin presented an additional difficulty because of the low proportion of melanocytes (Weinstein et al., 1991; Schwindinger et al., 1992; Lumbroso et al., 2004; Vasilev et al., 2014). In our series, this hypothesis is confirmed by the unexpected results obtained in two intriguing cases presenting discrepancies between blood and cutaneous biopsies. In patient 41, the cutaneous biopsy resulted in negative but blood-extracted DNA was positive for a *GNAS* mutation. An artifact was excluded as we confirmed this positive result in two independently collected and extracted blood samples by both dPCR and COLD-MAMA PCR. The best explanation for this finding is the coring of a tissue mainly composed of normal cells. This is further supported by our findings in patient 43 where a mutated allele was detected both in blood and in cultured fibroblasts but not in the skin.

This work included a wide and heterogeneous case series, reflecting different clinical presentations, as well as various sample types. Even if a negative genetic investigation does not rule out the clinical diagnosis of MAS/FD, the detection of pathogenetic variants associated with MAS/FD is fundamental, in particular in partial forms, as it allows early diagnosis.

In conclusion, our data clearly confirm the difficulty encountered in performing a successful molecular diagnosis of *GNAS* somatic activating variants, even from tissue specimens. The strengths of the dPCR approach were 1) being less susceptible to poor quality sample issues (short amplicons), leading to successful amplification also of degraded DNA; 2) being less sensitive to the presence of inhibitors (sample dilution); 3) very low associated risk of cross-contamination (no preamplification cycles required); and 4) being cost-effective (1-day processing time and appropriate cost of reagent). Overall, the dPCR demonstrated to be a sensitive, accurate, and specific analytical tool that we propose should be used as the first-line choice for the molecular diagnosis of MAS/FD.

## Data Availability

Data generated and/or analyzed during this study are included in this published article, and they are available from the corresponding author on reasonable request.

## Ethics Statement

Informed consent was obtained from all patients (or legal guardians for minors) and relatives included in the present study. All procedures were performed in compliance with relevant legislation and institutional guidelines and were approved by the IRCCS Fondazione CàGrandaOspedale Maggiore Policlinico institutional committee.

## Author Contributions

FE conceived and designed the project, analyzed and interpreted data, and was a major contributor in writing the manuscript. MB, IG, MM, AP, and PB acquired and analyzed data. GM conceived and designed the project, followed patients, interpreted data, and was a major contributor in writing the manuscript. LS and MA followed patients and were a minor contributor in writing the manuscript. All authors read and approved the final manuscript.

## Funding

This work was supported by the Italian Ministry of Health under Grant GR-2009-1608394; Fondazione IRCCS Ca’ Granda Policlinico Ospedale Maggiore under Grant Ricerca Corrente Funds.

## Conflict of Interest Statement

The authors declare that the research was conducted in the absence of any commercial or financial relationships that could be construed as a potential conflict of interest.
