# Supplementary material for: Improved Molecular Diagnosis of McCune–Albright Syndrome and Bone Fibrous Dysplasia by Digital PCR
Source: Front Genet. 2019 Sep 18;10:862. doi: 10.3389/fgene.2019.00862 (PMC6760069; doi:10.3389/fgene.2019.00862)
Supplement: Supplementary file 1 [file Table_1.docx]

**Supplementary table 1.** Table resuming clinical features of the studied cohort of MAS patients. Legend: diag.age, molecular diagnosis age; FD, bone fibrous dysplasia; PP, precocious puberty; SP, cafè-au-lait pigmented skin lesions; add. clin. features, additional clinical features; F, female; M, male; WT, wild-type; MUT, mutated.

| **PT ID** | **SEX** | **DIAG.AGE** | **FD** | **PP** | **OC** | **TL** | **SP** | **HT** | **GH** | **nHC** | **Add. Clin. features** | **c.604C>T genotype** | **c.605G>A genotype** |
| --- | --- | --- | --- | --- | --- | --- | --- | --- | --- | --- | --- | --- | --- |
| **1** | F | 43 | X |  |  |  |  |  |  |  |  | WT | **MUT** |
| **2** | F | 8 | X |  |  |  |  |  |  |  |  | WT | **MUT** |
| **3** | M | 13 | X |  |  |  |  |  |  |  |  | WT | **MUT** |
| **4** | F | 22 | X |  |  |  |  |  |  |  |  | WT | WT |
| **5** | F | 18 | X |  |  |  |  |  |  |  | Hodgkin's lymphoma | WT | **MUT** |
| **6** | F | 11 | X |  |  |  |  |  |  |  |  | WT | **MUT** |
| **7** | F | 53 | X |  |  |  |  |  |  |  |  | WT | WT |
| **8** | F | 34 | X |  |  |  |  |  |  |  |  | WT | WT |
| **9** | F | 11 | X |  |  |  | X |  |  |  |  | WT | WT |
| **10** | M | 5 | X |  |  |  | X | X |  |  |  | WT | WT |
| **11** | F | 4 | X |  |  |  |  |  |  |  |  | WT | WT |
| **12** | M | 22 | X |  |  |  |  |  |  |  |  | WT | **MUT** |
| **13** | F | 1 |  | X |  |  | X |  |  |  |  | WT | WT |
| **14** | F | 22 |  |  | X |  | X |  |  |  | osseous cyst | WT | WT |
| **15** | F | 12 |  | X |  |  | X |  |  |  | short stature - bilateral genu valgum -bilateral clinodactyly | WT | WT |
| **16** | F | 2 |  | X |  |  | X |  |  |  |  | WT | WT |
| **17** | M | 10 |  | X |  |  |  |  |  |  | psychomotor delay - bilateral testicular calcifications | WT | WT |
| **18** | M | 6 |  | X |  |  | X |  |  |  | gynecomastia | WT | WT |
| **19** | M | 2 | X |  |  |  | X |  |  |  |  | WT | **MUT** |
| **20** | F | 6 |  |  |  |  | X |  |  |  | adrenal tumor - clitoral hypertrophy | WT | WT |
| **21** | F | 12 |  | X |  |  | X |  |  |  |  | WT | WT |
| **22** | F | 3 |  |  |  |  | X | X | X |  | hyperchromic nevus | WT | **MUT** |
| **23** | F | 3 |  | X | X |  |  |  |  |  |  | WT | WT |
| **24** | F | 12 | X |  |  |  | X |  |  |  |  | WT | WT |
| **25** | M | 7 | X |  |  |  |  |  |  |  |  | WT | **MUT** |
| **26** | F | 11 | X |  |  |  |  |  |  |  |  | WT | WT |
| **27** | F | 2 |  | X |  |  |  |  |  |  | muscular hypotonia, absent language | WT | WT |
| **28** | F | 9 |  | X |  |  | X |  |  |  |  | WT | **MUT** |
| **29** | F | 6 |  | X |  |  | X |  |  |  | advanced bone age | WT | WT |
| **30** | F | 1 |  | X |  |  |  |  |  |  |  | WT | WT |
| **31** | F | 2 | X |  |  |  | X |  |  |  |  | WT | WT |
| **32** | M | 14 |  |  |  |  | X |  |  |  | overweight - mild sindactyly | WT | WT |
| **33** | F | 2 |  | X |  |  | X |  |  |  |  | WT | WT |
| **34** | M | 46 | X |  |  |  |  |  |  |  |  | WT | WT |
| **35** | F | 2 |  | X | X |  |  |  |  |  |  | WT | WT |
| **36** | M | 2 | X |  |  |  |  |  |  |  |  | WT | WT |
| **37** | M | 56 | X |  |  |  |  |  |  |  |  | WT | **MUT** |
| **38** | F | 3 |  | X | X |  | X |  |  |  |  | WT | WT |
| **39** | M | 12 |  |  |  |  | X |  |  |  |  | WT | **MUT** |
| **40** | F | 5 |  | X |  |  | X |  |  |  | hypovitaminosis D | WT | WT |
| **41** | F | 48 |  |  |  |  | X |  |  |  |  | **MUT** | WT |
| **42** | M | 15 | X |  |  |  |  |  |  |  |  | WT | WT |
| **43** | F | 11 | X | X |  |  | X |  |  |  |  | WT | **MUT** |
| **44** | F | 6 |  | X | X |  | X |  |  |  |  | **MUT** | WT |
| **45** | M | 17 | X |  |  |  |  |  |  |  |  | WT | WT |
| **46** | F | 6 |  | X | X |  |  |  |  |  |  | WT | **MUT** |
| **47** | F | 1 | X | X | X |  | X |  |  |  | hypophosphoremia | WT | **MUT** |
| **48** | F | 6 | X | X | X |  |  |  |  |  |  | WT | **MUT** |
| **49** | F | 5 | X | X | X |  | X |  |  |  |  | WT | **MUT** |
| **50** | F |  |  | X | X |  |  |  |  |  |  | WT | **MUT** |
| **51** | M | 9 | X |  |  |  | X |  |  |  |  | WT | **MUT** |
| **52** | M | 1 |  |  |  |  | X |  |  |  | intrahepatic cholestasis - posterior embriotoxon | WT | **MUT** |
| **53** | F | 2 | X |  |  |  |  |  |  |  |  | WT | **MUT** |
| **54** | F | 46 | X |  |  |  |  |  |  |  |  | WT | WT |

**Supplementary table 2.** Table resuming the cluster of MAS mutated patients. BO, bone. BL, blood. LJ, left jaw. M, maxilla. RJ, right jaw, S, symphysis. CB, cutaneous biopsy. FB, fibroblast. OCL, ovarian cyst liquid. OC, ovarian cyst. OT, ovarian tissue. nd, not determined because methods failed due to the reduced amount or the poor quality of material available for testing. WT, wild-type.

|  |  | **c.604C>T** | | | | | | | **c.605G>A** | | | | | | |
| --- | --- | --- | --- | --- | --- | --- | --- | --- | --- | --- | --- | --- | --- | --- | --- |
| **PT ID** | **TISSUE** | **SANGER** | **AS-PCR** | **dPCR** | **dPCR cpm** | **dPCR RMA** | **COLD-MAMA PCR** | **COLD-MAMA RMA** | **SANGER** | **AS-PCR** | **dPCR** | **dPCR cpm** | **dPCR RMA** | **COLD-MAMA PCR** | **COLD-MAMA RMA** |
| **1** | **BO** | WT | WT | WT | 0.181 |  |  |  | WT | WT | **MUT** | **0.892** | **>6** |  |  |
| **2** | **BO** | WT | WT | WT | 0.000 |  |  |  | **MUT** | **MUT** | **MUT** | **8.712** | **>50** |  |  |
| **3** | **BO** | WT | WT | WT | 0.095 |  |  |  | **MUT** | **MUT** | **MUT** | **17.626** | **100** |  |  |
| **5** | **BO** | WT | nd | WT | 0.000 |  | nd |  | WT | nd | **MUT** | **6.594** | **50** | nd |  |
| **6** | **BO** | WT | WT | WT | 0.000 |  | WT |  | WT | **MUT** | **MUT** | **3.604** | **25** | **MUT** | **3** |
| **12** | **BO** | WT | nd | WT | 0.260 |  | nd |  | WT | nd | **MUT** | **3.196** | **25** | nd |  |
| **19** | **BL** | WT | WT | WT | 0.117 |  | WT |  | WT | WT | **MUT** | **0.815** | **>6** | WT |  |
| **22** | **BL** | WT | WT | WT | 0.000 |  |  |  | WT | WT | **MUT** | **0.644** | **6** |  |  |
| **25** | **BL** | WT | WT | WT | 0.000 |  | nd |  | WT | WT | **MUT** | **2.252** | **>12.5** | nd |  |
| **28** | **BL** | WT | WT | WT | 0.000 |  | nd |  | WT | **MUT** | **MUT** | **2.170** | **>12.5** | nd |  |
| **51** | **BL** | WT | nd | WT | 0.000 |  | nd |  | WT | nd | **MUT** | **1.216** | **12.5** | nd |  |
| **53** | **BL** | WT | nd | WT | 0.258 |  | nd |  | WT | nd | **MUT** | **1.043** | **<12.5** | nd |  |
| **37** | **BL** | WT | WT | WT | 0.166 |  | WT |  | WT | WT | **MUT** | **0.622** | **>6** | **MUT** | **6** |
|  | **BO-LJ** | WT | WT | WT | 0.195 |  | WT |  | **MUT** | **MUT** | **MUT** | **32.654** | **100** | **MUT** | **>40** |
|  | **BO-M** | WT | nd | WT | 0.000 |  |  |  | **MUT** | nd | **MUT** | **22.108** | **100** | nd |  |
|  | **BO-RJ** | WT | WT | WT | 0.169 |  | WT |  | WT | **MUT** | **MUT** | **2.121** | **<25** | **MUT** | **3** |
|  | **BO-S** | WT | nd | WT | 0.188 |  |  |  | **MUT** | nd | **MUT** | **22.904** | **100** | nd |  |
| **39** | **BL** | WT | WT | WT | 0.319 |  |  |  | WT | WT | **MUT** | **0.509** | **6** |  |  |
|  | **CB** | WT | WT | WT | 0.000 |  |  |  | WT | WT | **MUT** | **1.251** | **<12.5** |  |  |
| **52** | **BL** | WT | WT | WT | 0.112 |  | nd |  | WT | **MUT** | **MUT** | **0.598** | **6** | nd |  |
|  | **CB** | WT | WT | WT | 0.000 |  | nd |  | WT | **MUT** | **MUT** | **6.208** | **50** | nd |  |
| **41** | **BL** | WT | WT | **MUT** | **2.249** | **<12.5** | **MUT** | **<2.5** | WT | WT | WT | 0.254 |  | WT |  |
| **43** | **BL** | WT | WT | WT | 0.188 |  |  |  | WT | WT | **MUT** | **0.873** | **>6** |  |  |
|  | **FB** | WT | WT | WT | 0.000 |  |  |  | WT | WT | **MUT** | **1.633** | **>12.5** |  |  |
| **44** | **BL** | WT | WT | **MUT** | **0.777** | **<3** | WT |  | WT | WT | WT | 0.293 |  | WT |  |
|  | **OCL** | WT | WT | **MUT** | **4.995** | **<25** | **MUT** | **<2.5** | WT | WT | WT | 0.256 |  | WT |  |
|  | **OT** | WT | WT | **MUT** | **1.485** | **>6** | WT |  | WT | WT | WT | 0.184 |  | WT |  |
| **46** | **OC** | WT | WT | WT | 0.083 |  | WT |  | **MUT** | **MUT** | **MUT** | **2.969** | **<25** | **MUT** | **10** |
|  | **OT** | WT | WT | WT | 0.182 |  | WT |  | WT | WT | **MUT** | **0.520** | **6** | **MUT** | **<0.5** |
| **47** | **OC** | WT | WT | WT | 0.102 |  | WT |  | WT | WT | **MUT** | **1.135** | **<12.5** | WT |  |
|  | **OT** | WT | WT | WT | 0.084 |  | WT |  | WT | WT | **MUT** | **1.164** | **<12.5** | **MUT** | **3** |
| **48** | **BL** | WT | WT | WT | 0.334 |  | WT |  | WT | **MUT** | **MUT** | **5.347** | **<50** | **MUT** | **16** |
|  | **OC** | WT | WT | WT | 0.000 |  | WT |  | WT | **MUT** | **MUT** | **10.169** | **>50** | **MUT** | **>40** |
| **49** | **BL** | WT | WT | WT | 0.101 |  | WT |  | WT | WT | **MUT** | **0.465** | **3** | WT |  |
|  | **OC** | WT | WT | WT | 0.192 |  | WT |  | WT | WT | **MUT** | **1.578** | **>12.5** | **MUT** | **26** |
| **50** | **OC** | WT | WT | WT | 0.412 |  | WT |  | WT | **MUT** | **MUT** | **3.312** | **25** | **MUT** | **<0.5** |
|  | **OCL** | WT | WT | WT | 0.205 |  | WT |  | WT | **MUT** | **MUT** | **7.275** | **>50** | **MUT** | **2.5** |
